# Dissipative Particle Dynamic Simulation on Self-Assembly of Symmetric CBABC Pentablock Terpolymers in Solution

**DOI:** 10.3390/ma16237273

**Published:** 2023-11-22

**Authors:** Yingying Guo

**Affiliations:** School of Science, Qingdao University of Technology, Qingdao 266525, China; gyyhappy@outlook.com

**Keywords:** phase behavior, pentablock terpolymers, self-assembly, onion-like vesicle, unilamellar vesicle

## Abstract

Pentablock terpolymers are potential candidates for the self-assembly of multicompartment nanostructures. In this work, Dissipative Particle Dynamic simulation is employed to investigate how the equilibrium aggregate structures of C_3_B_3_A_6_B_3_C_3_ pentablock terpolymers are affected by polymer–solvent interactions in a solution. Multicompartment structures, such as layered micelles, onion-like micelles, onion-like vesicles, unilamellar vesicles, and vesicle-in-vesicle structures, are observed. Vesicles are obtained when the two end C-blocks or the central A-block are hydrophilic. The solvent encapsulation ability and vesicle membrane permeability are assessed. The unilamellar vesicle shows higher encapsulation efficiency and lower membrane permeability compared with the onion-like vesicles. Additionally, the two vesicles show different responses to shear. While the cargo release rate of the onion-like vesicle is not affected by shear, shear results in a slowdown of the release rate for the unilamellar vesicle. The membrane thickness of the unilamellar vesicle can be adjusted using the length of the central A-blocks. Vesicles with thicker membranes hold cargo more effectively.

## 1. Introduction

Amphiphilic block copolymers are a class of polymers that contain both solvophilic (solvent-loving) and solvophobic (solvent-repelling) segments. In the presence of a selective solvent, amphiphilic block copolymers exhibit the capability to undergo self-assembly, resulting in diverse morphologies such as micelles and vesicles. The inherent self-assembly potential of amphiphilic block copolymers in selective solvents has found applications across various domains in both fundamental and applied sciences, including diagnostics [1,2], drug delivery [3,4], and chemical sensing [5,6]. As nanoscience continues to evolve, advanced micellar systems, characterized by multicompartment structures and hierarchical organization, will continue to garner significant interest [7].

The self-assembled structures are influenced by various factors, including the length of each block, the number of blocks, the overall molecular weight of the copolymer, preparation conditions, the nature of the selective solvent, and molecular architecture. Recently, researchers have expanded the diversity and functionality of nanostructures formed by self-assembling block copolymers by increasing the number of blocks and introducing additional block types [8,9]. These block copolymers are referred to as multiblock copolymers. Nevertheless, investigations into the self-assembly of multiblock copolymers in solutions are still at an early stage, and the observation of micelles with multicompartment nanostructures remains rare [7]. The symmetric ABCBA linear pentablock terpolymer with a block number of 5 has been considered a representative model and a starting point for studying the self-assembly of multiblock copolymers [10].

Efforts have been dedicated to studying the self-assembly behavior of amphiphilic pentablock terpolymers under selective solutions [7,11,12,13,14,15,16]. When thermosensitive blocks were introduced to the two free ends of pentablock terpolymers, Lv et al. observed transformations in self-assembly morphologies in response to the Lower Critical Solution Temperature (LCST) [16]. Consequently, they obtained core–corona spheres, core–corona cylinders, core–shell–corona spheres, and large compound spheres. Gao et al. [12] synthesized a series of pentablock terpolymers, PAAx-b-PS48-b-PEO46-b-PS48-b-PAAx, with varying lengths of PAA blocks and investigated their self-assembly behavior. As the length of PAA increased, the micelle morphology underwent a series of transformations, beginning with spherical vesicles featuring ordered porous membranes. The morphology then transitioned to long double nanotubes, followed by long nanotubes with inner modulated nanotubes or short nanotubes. Ultimately, the morphology evolved into spherical micelles or large compound vesicles containing spherical micelles.

Among these self-assembled morphologies, vesicles are considered good candidates for targeted release. In most applications [17,18,19,20], unintentional leaks should be prevented. However, in some cases, carriers with more permeable membranes are desired to enable core/shell transport [21,22]. Cargo release from a vesicle could occur when external factors induce changes in the membrane permeability of a vesicle. These external factors may include temperature [23,24,25,26], pH value [27,28,29,30], light [31,32], and magnetic fields [33]. In a study conducted by Popescu et al. [13], an investigation into the self-assembly of an amphiphilic pentablock terpolymer was carried out. This terpolymer included a pH-sensitive poly(2-vinylpyridine) (P2VP) central block. The resulting vesicles displayed a distinctive “flower-like” morphology, characterized by a membrane composed of self-assembled hydrophobic P2VP central blocks. The study demonstrated the ability to modulate the release profile of these vesicles by adjusting membrane permeability through chemical means (such as partial quaternization) and/or physical methods (including pH variations).

Shear [30,34,35,36] is also a significant external stimulus for cargo release. Rifaie-Graham et al. [37] modified amphiphilic block copolymers by incorporating a small percentage of nucleobases into their hydrophobic block. When shear stress is applied, nucleobase pairs in the hydrophobic leaflet separate and expose their hydrogen bonding motifs, rendering the membrane more permeable to water-soluble compounds. Shear is involved in many industrial processes. Research on vesicles has primarily concentrated on biological applications; however, the mechanical stability of polymeric vesicles introduces opportunities for their application in engineering. This is especially relevant in situations where targeted cargo delivery can offer distinct advantages.

This paper investigates the self-assembly behavior of CBABC pentablock terpolymers in selective solutions using Dissipative Particle Dynamic (DPD) simulations. In comparison to recent research [14], which mainly focuses on the effects of block sequence, block composition, and polymer concentration on chain conformation and self-assembled morphology, our research is centered on exploring the effects of solvent selectivity toward either the two free-end C-blocks or the central A-block. Given that vesicles formed from these pentablock terpolymers can serve as efficient carriers, comprehending how their encapsulation efficiency and permeability are influenced by their morphological characteristics is essential. In our previous work [36], we investigated the shear release behavior of vesicles made of ABC star terpolymers, ABA star terpolymers, and AB linear copolymers. All the vesicles exhibited a slow-release rate under bulk shear. The cargoes were found to be released through the membranes of the vesicles. While pentablock terpolymers are reported to have improved mechanical properties [38], the mechanical behavior of vesicles made of pentablock terpolymers is still unknown. Building on this, we have also investigated cargo release mechanisms under shear conditions.

## 2. Simulation Methods

### 2.1. Dissipative Particle Dynamic Method

We employed Dissipative Particle Dynamic (DPD) simulations [39] to investigate the phase behavior of CBABC pentablock terpolymers in a selective solution. DPD is a particle-based mesoscopic simulation technique where polymers are coarse-grained and made up of DPD particles of equal size. The motion of each DPD particle is governed by Newton’s equation of motion. The total force exerted on particle i, denoted as ${\vec{f}}_{i\;}$, comprises four components. The three components, i.e., the conservative force ${\vec{F}}_{ij}^{C}$, dissipative force ${\vec{F}}_{ij}^{D}$, and random force ${\vec{F}}_{ij}^{R}$, contribute in a pairwise manner and become effective when the distance between two beads i and j is within the cut-off radius $r_{c}$. The conservative force ${\vec{F}}_{ij}^{C}$ is a soft-repulsive force and is given by

(1) $${\vec{F}}_{ij}^{C}=\left\{\begin{array}{c} a_{ij}\left(1-r_{ij}/r_{c}\right){\hat{r}}_{ij}\left(r_{ij}<r_{c}\right)\\ \;0\;\left(r_{ij}>r_{c}\right) \end{array}\right.$$

where ${\vec{r}}_{ij}={\vec{r}}_{i}-{\vec{r}}_{j}$*,* $r_{ij}=\left|{\vec{r}}_{ij}\right|$, ${\hat{r}}_{ij}=1/\left|{\vec{r}}_{ij}\right|{\vec{r}}_{ij}$, $a_{ij}$ represents the maximum repulsion between beads i and j, $r_{c}$ is the cut-off radius with a value of 1.0, and $a_{ij}$ is chosen according to the linear relationship with Flory−Huggins χ parameter proposed by Groot and Warren [40], where $a_{ij}=a_{ii}+3.27\chi_{ij}$. For a system with density ρ=3, $a_{ii}=25$. The combination of dissipative force ${\vec{F}}_{ij}^{D}$ and random force ${\vec{F}}_{ij}^{R}$ acts as a thermostat in the simulation. The two forces are evaluated as

(2) $${\overset{⃑}{F}}_{ij}^{D}=-\gamma \omega^{D}\left(r_{ij}\right){\overset{⃑}{v}}_{ij}\cdot {\overset{⃑}{r}}_{ij}{\hat{r}}_{ij}$$

(3) $${\overset{⃑}{F}}_{ij}^{R}=-\sigma \omega^{R}\left(r_{ij}\right)\frac{\xi_{ij}}{\sqrt{\Delta t}}{\hat{r}}_{ij}$$

where ${\overset{⃑}{v}}_{ij}={\overset{⃑}{v}}_{i}-{\overset{⃑}{v}}_{j}$; σ and γ, respectively, represent the amplitude of ${\overset{⃑}{F}}_{ij}^{D}$ and ${\overset{⃑}{F}}_{ij}^{R}$; $\omega^{D}\left(r_{ij}\right)$ and $\omega^{R}\left(r_{ij}\right)$ are the weight functions; and $\xi_{ij}$ is a random number with zero mean and unit variance. In order to satisfy the equilibrium Gibbs–Boltzmann distribution and the fluctuation-dissipative theorem, the following two relationships are required:

(4) $$\omega^{D}\left(r_{ij}\right)={\left(\omega^{R}\left(r_{ij}\right)\right)}^{2}$$

(5) $$\sigma^{2}=2\gamma k_{b}T$$

In our simulations, σ was chosen to be 3 [40] and temperature $k_{B}T$ was chosen to be 1. According to Groot and Warren [40], the weight function is expressed as

(6) $$\omega^{D}\left(r_{ij}\right)=\left\{\begin{array}{c} {\left(1-\frac{r_{ij}}{r_{c}}\right)}^{2}\;(r_{ij}<r_{c})\\ \;0\;\left(r_{ij}\geq r_{c}\right) \end{array}\right.$$

A spring force ${\vec{F}}_{ij}^{S}$ is then introduced between beads connected by covalent bonds to simulate polymer chains. The force is given by

(7) $${\overset{⃑}{F}}_{ij}^{S}=\sum_{j}C(r_{ij}-r_{eq}){\hat{r}}_{ij}$$

where C is the spring constant and set as 8.0 and the equilibrium bond length is $r_{eq}=0$ [40]. The numerical integration of the equation of motion was conducted using the Velocity Verlet algorithm with a time step of ∆t=0.04 in DPD units.

### 2.2. Polymer Model: Simulation Conditions

**Figure 1:**
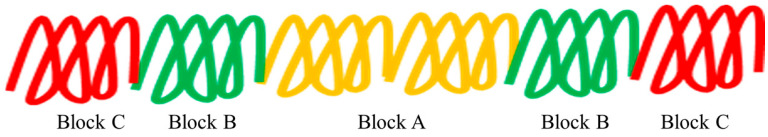
Schematic showing the model of linear symmetric CBABC pentablock terpolymers.

The model systems consist of symmetric pentablock terpolymers composed of A-, B-, and C-beads in a solvent, denoted as S. The schematic representation of our model, the CBABC pentablock terpolymer chain, is depicted in Figure 1. In the figure, C-blocks are positioned at both free ends of the chain, while the A-block is located in the middle part of the chain. The A-, B-, and C-blocks are color-coded as yellow, green, and red, respectively. In the work, the C_3_B_3_A_6_B_3_C_3_ pentablock terpolymers are chosen as the simulation model, with the exception of an additional statement we make.

Given the vast phase space of the polymer system in the presence of a solvent, we have simplified our simulation. All interaction parameters among polymer beads are set to be equal, i.e., $a_{AB}=a_{BC}=a_{AC}=40$. This corresponds to weak segregation strength among polymer blocks. The interaction parameter between B-block and the solvent is set at $a_{BS}=45$. Since our focus is on the impact of the solvophobicity of the two end C-blocks and middle A-block on the self-assembly behavior, we varied the interaction parameter between the free end C-blocks (middle A-block) and the solvent within the range of 26 to 105. The interaction parameters are listed in Table 1.

The polymer volume fraction ($\varphi_{P}$) is determined by dividing the total number of polymer beads by the overall number of beads in the simulations. For all simulations, a polymer volume concentration $\varphi_{p}=$ 10% is used. The simulation begins with a homogenous state, and a minimum of 2 million DPD steps are taken to ensure equilibrium states are achieved. We employ the NVT ensemble in the simulation.

We applied bulk shear to investigate the structural stability and cargo release behavior of vesicles. This shear was induced by altering the shape of the simulation box during the dynamic run. We applied a constant strain rate of $\dot{\gamma }=0.001$ DPD unit. The strain was calculated by dividing the offset by the length. Here, the length corresponds to the initial box length in the direction perpendicular to shear (e.g., the z box length for xz deformation), and the offset represents the change in the shear direction (e.g., the alteration in the x box length for xz deformation) [36].

## 3. Results and Discussion

### 3.1. State Diagram and General Description of Equilibrium Aggregate Structure

**Figure 2:**
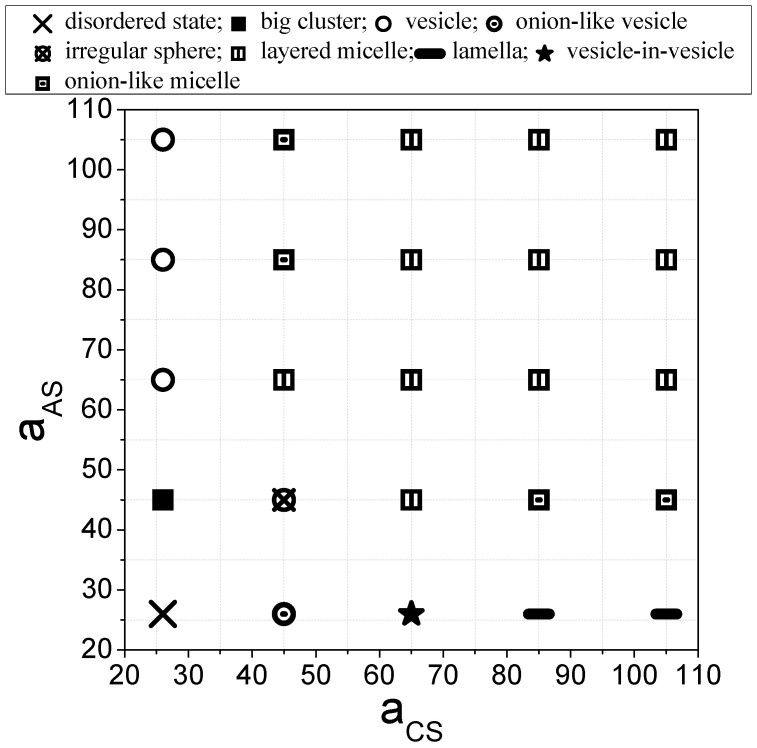
The effect of the interaction energies between the A-block and the solvent, as well as between the C-block and the solvent, on the equilibrium aggregate structures.

To illustrate the effect of the interaction strength of the two end C-blocks and central A-blocks with the solvent, we present equilibrium aggregate structures for various value of $a_{AS}$ and $a_{CS}$ in Figure 2, shown in the form of a state diagram. As depicted in Figure 2, the diagram exhibits an A-C reflection symmetric at $a_{AS}$ and $a_{CS}$ values greater than 26. To facilitate a more detailed analysis of the morphology transition, we divide the phase diagram into four main regions: (a) both $a_{AS}$ and $a_{CS}$ larger than $a_{BS}$; (b) $a_{AS}=a_{BS}\leq a_{CS}$ or $a_{BS}=a_{CS}\leq a_{AS}$; (c) $a_{AS}=26$; and (d) $a_{CS}=26$.

**Figure 3:**
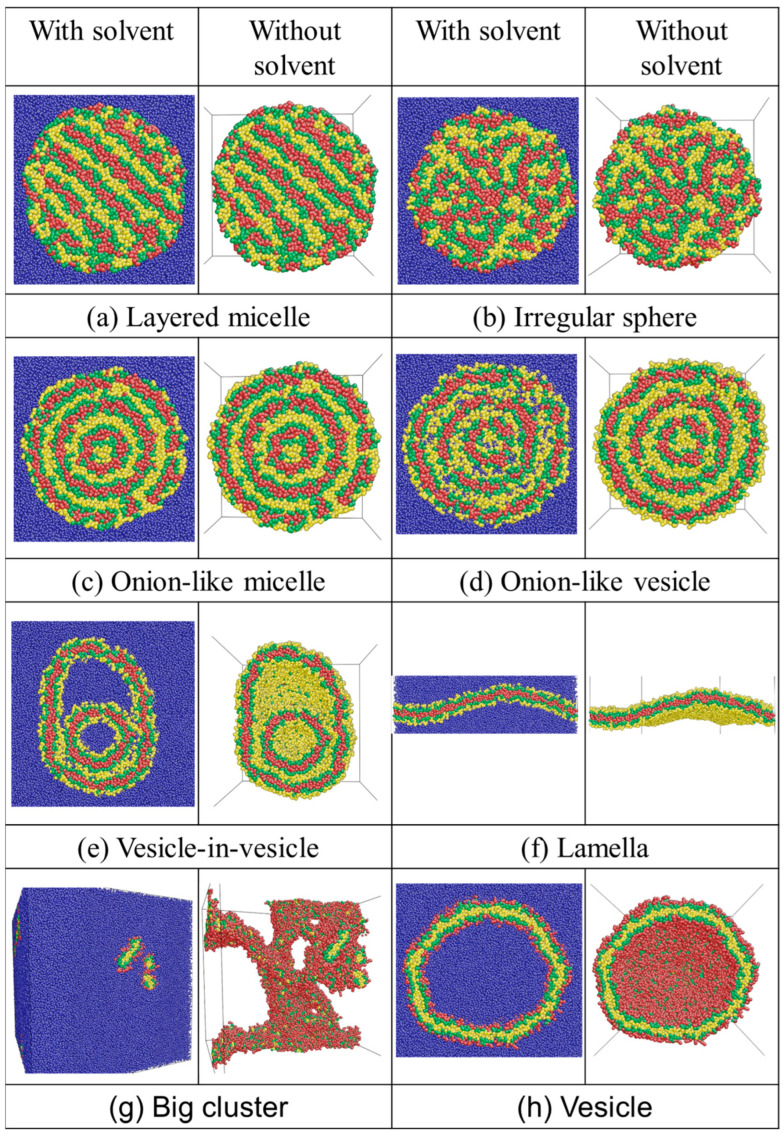
Typical snapshots for the self-assembled structures of CBABC pentablock terpolymers in the solvent at different solvent-block interactions. Yellow, green, red, and blue beads correspond to A-, B-, C-, and S-beads, respectively.

The typical equilibrium morphologies are shown in Figure 3. In region (a), where the solvent beads exhibit a preference for B-blocks, spherical layered micelles (◫ in Figure 2) are observed. These micelles consist of an outer shell and an inner core. The shell of an aggregate consists of A-, B- and C-beads which are in contact with S-beads. Meanwhile, the inner core of the micelle exhibits distinct layers of A-, B-, and C-beads with parallel alignment to each other (as shown in Figure 3a).

**Figure 4:**
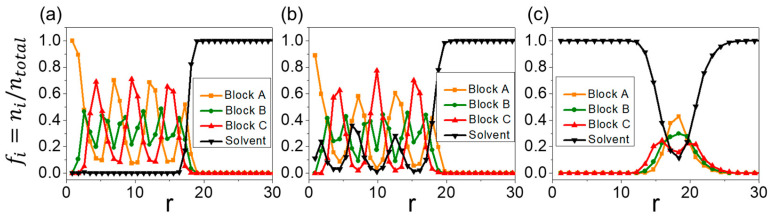
Composition profiles for (**a**) multilayer micelle, (**b**) multilayer vesicle, and (**c**) vesicle. The horizontal axis represents the distance from the center of mass of the aggregate to the position of the bead, while the vertical axis denotes the number fraction of bead i at a specific r. The fraction is calculated as the ratio of the number of beads of type i to the total number of beads at a specific distance from the center of mass.

In region (b), when $a_{AS}=a_{BS}=a_{CS}=45$, meaning that the solvent has equal repulsion to all blocks, an irregular sphere (⦻ in Figure 2) is obtained. This structure consists of small clusters of A-, B-, and C-beads randomly packed into a spherical morphology (Figure 3b). When $a_{CS}$ or $a_{AS}$ increases to 65, the inner core of the irregular-sphere micelle phase separates into layered structures. A further increase in $a_{CS}$ or $a_{AS}$ results in the formation of three-lamellae onion-like micelles (▣ in Figure 2; Figure 3c). To better understand the structures, composition profiles are presented in Figure 4a. These profiles show how fractions of different beads $f_{i}$ (i = A, B, C, and S) vary from the center of mass of the micelles to their peripheries. Notably, no solvent beads are encapsulated inside the structure. The composition profile reveals a concentric lamellar structure characterized by alternating peaks of A-, B-, and C-blocks.

Next, we move to region (c) and region (d). At the intersection of these two regions, where interactions among all beads and the solvent are relatively weak, the solvent and the terpolymer blend well, resulting in a disordered state (indicated by ✕ in Figure 2). In this state, most terpolymer chains remain as individual chains. An increase in either $a_{CS}$ (region (c)) or $a_{AS}$ (region (d)) promotes phase separation driven by repulsion among the beads.

At $a_{AS}=26$ (region (c)), where the solvent prefers the central block of the chain, morphologies transition to onion-like vesicles (⊙ in Figure 2) as $a_{CS}$ increases to 45. As shown in Figure 3d, the structure exhibits concentric onion-like layers consisting of distinct A-, B-, and C-layers. This structure shares similarities with concentric micelle structures; however, in the concentric vesicle, solvent beads can be observed within the hydrophilic layers primarily made of A-beads. This observation is confirmed by the composition profile, where S-peaks correspond with A-peaks. At $a_{CS}$ = 65, a structure-in-structure morphology emerges. The morphology comprises a small bilayer vesicle embedded inside a larger bilayer vesicle (★ in Figure 2; Figure 3e). A significant portion of solvent beads (blue) is encapsulated within the both vesicles.

As $a_{CS}$ is further increased to 105, where C-blocks exhibit strong repulsion to the solvent, a planar lamella phase (– in Figure 2; Figure 3f) is observed. The planar lamella phase displays a bilayer membrane structure, with a hydrophobic inner leaflet of the membrane primarily made of C-beads (red). The inner leaflet is shielded by layers formed with B-beads. The hydrophilic A-beads form the bottom and upper outer shells to cover the layers, preventing unfavorable interactions between B- and C-blocks and the solvent.

At $a_{CS}=26$ (region (d)), where the solvent favors the two free ends of the chain, an irregular large cluster (■ in Figure 2; Figure 3g) is observed at $a_{AS}=45$. The morphologies then transform into vesicles (○ in Figure 2; Figure 3h) as $a_{AS}$ increases, while $a_{CS}$ remains smaller than $a_{BS}$ and $a_{AS}$. These vesicles encapsulate S-beads (blue) within their bilayer membranes (Figure 3e). The composition profile could be seen in Figure 4c. The vesicle membranes consist of a hydrophobic inner leaflet made primarily of A-beads (yellow). This inner leaflet is shielded by layers formed with B-beads (green). The hydrophilic C-beads (red) aggregate on both the inner and outer shells of the membrane. In addition to the vesicle’s cavity, a small portion ($f_{i}\approx 0.1$) of solvent beads is observed to cross the vesicle’s membrane.

In short, multicompartment micelles, including layered micelles and onion-like micelles, are obtained in regions where the interaction between the solvent and the two end C-blocks and the interaction between the solvent and the central A-block are stronger than the interaction between the solvent and the B-blocks (i.e., $a_{AS}\geq a_{BS}$ and $a_{CS}\geq a_{BS}$). Moreover, three types of vesicles are observed. Unilamellar vesicles are obtained when the two free end C-blocks are hydrophilic and $a_{AS}>a_{BS}$. Onion-like vesicles are obtained when the central A-blocks are hydrophilic and $a_{BS}=a_{CS}$. The vesicle-in-vesicle structures are obtained when the central A-blocks are hydrophilic and $a_{CS}>a_{BS}$.

### 3.2. Formation Mechanism of Onion-like Aggregates

Onion-like aggregates possess a multilayer membrane structure, offering the potential for precise targeted release by encapsulating the same or different cargoes in each membrane layer. In our system, we observe two types of onion-like aggregates: onion-like micelles and onion-like vesicles. To gain a deeper understanding of these two structures, we investigate their formation mechanisms.

**Figure 5:**
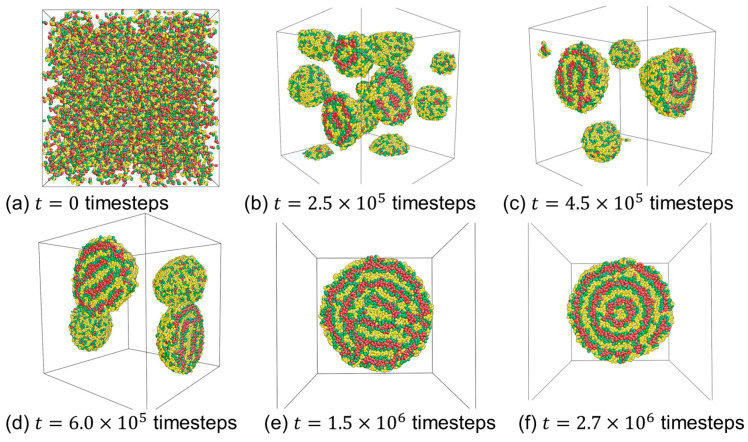
Dynamic formation of the onion-like micelle formed by the self-assembly of CBABCA pentablock terpolymers in a solvent at $a_{AS}=45\;and{\;a}_{CS}=85$. Red, green, and yellow beads correspond to *C*-, *B*-, and *A*-beads, respectively. Solvent beads are omitted for clarity.

Figure 5 illustrates the formation path of onion-like micelles at $a_{AS}=45,\;a_{CS}=85$. At the outset of the simulation, all the molecules are randomly dispersed within the simulation box (Figure 5a). Subsequently, these molecules quickly aggregate, forming numerous small spherical micelles (Figure 5b). Upon closer examination of the structure, it becomes evident that most of these spherical micelles possess a single layer made up of C-beads. These small spherical micelles gradually fuse to create larger spherical aggregates (Figure 5c,d), and within these aggregates, the micelles exhibit two layers comprising C-beads. This process continues as micelles merge, and some of them eventually evolve into large spherical micelles with fluctuating lamellar structures (Figure 5e). Finally, the micelles undergo a restructuring process, resulting in the formation of onion-like micelles, as shown in Figure 5f. Please note that the number of C-bead layers has been affected by the polymer concentrations [41]. For low concentrations, specifically 2% and 3%, micelles with a single C-bead layer and two C-bead layers have been observed.

**Figure 6:**
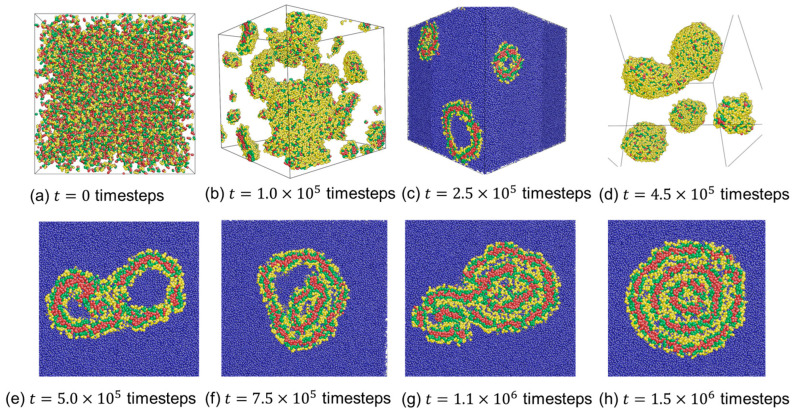
Dynamic formation of the onion-like vesicle formed by self-assembly of CBABC pentablock terpolymers in a solvent at $a_{AS}=26\;and\;a_{CS}=45$. Red, green, yellow, and blue beads correspond to *C*-, *B*-, *A*-, and *S*-beads, respectively. In some snapshots, solvent beads are omitted for clarity.

Figure 6 illustrates the structural evolution of an onion-like vesicle at $a_{AS}=26\;and\;a_{CS}=45$. Initially, polymer chains are randomly distributed in the solvent at timestep t=0 (Figure 6a). Owing to the solvophobicity of blocks B and C, these chains coalesce into small bilayer membranes and spherical micelles (Figure 6b). In the membrane structures, C-blocks form the inner leaflet, while the hydrophilic A-blocks constitute the outer shells of the membrane. These bilayer membranes gradually bend and eventually close to form spherical unilamellar vesicles (Figure 6c). Two vesicles then collide (Figure 6d). The outer hydrophilic layers of the two small vesicles unfold, and the hydrophobic layers subsequently make contact and fuse together, resulting in the formation of a vesicle-in-vesicle morphology (Figure 6e,f). These vesicle-in-vesicle structures further collide with the spherical micelles (Figure 6g) and restructure to form three-lamellar onion-like vesicles (Figure 6h).

### 3.3. Vesicle Membrane Permeability

Since the permeability of a vesicle membrane plays a crucial role in the ability of a vesicle to effectively isolate cargo from its environment, this section examines the membrane permeability of the two types of vesicles: unilamellar vesicle and onion-like vesicle. At the equilibrium state, the unilamellar vesicle encapsulates 43,733 cargoes, while the onion-like vesicle encapsulates 5389 cargoes.

**Figure 7:**
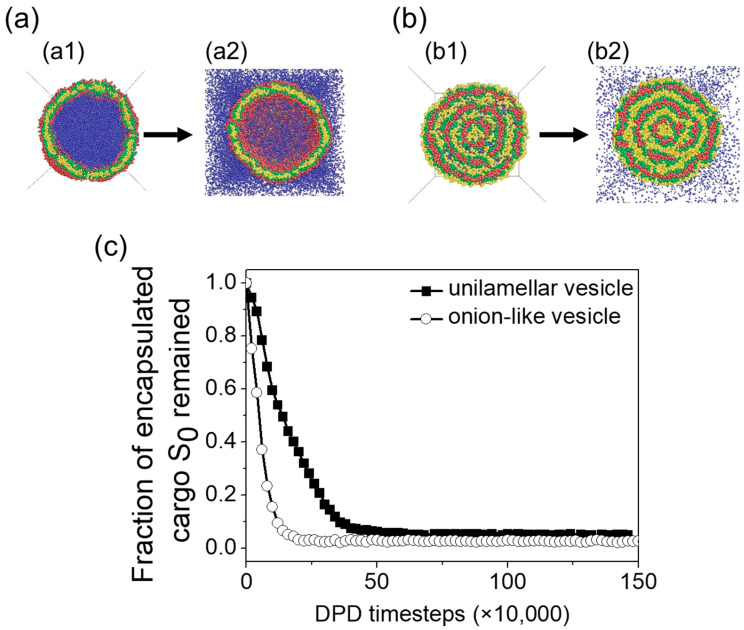
(**a**) Unilamellar vesicle structure at (**a1**) t=0 and (**a2**) $t=1.5\times 10^{6}$ timesteps; (**b**) onion-like vesicle structure at (**b1**) t=0 and (**b2**) $t=1.5\times 10^{6}$ timesteps; (**c**) the fraction of original $S_{0}$-beads encapsulated inside the vesicles. Red, green, yellow, and blue beads in (**a**,**b**) correspond to C-, B-, A-, and S-beads, respectively.

To assess membrane permeability, we placed the vesicles in the solvent and conducted a simulation for ${1.5\times 10}^{6}$ timesteps. The initial morphologies of the unilamellar vesicle and onion-like vesicle at t=0 are displayed in Figure 7a1,b1. For clarity, the cargo encapsulated inside the vesicles is marked in blue and denoted as $S_{0}$. Membrane permeability is characterized by the number of originally encapsulated cargoes that remain inside the vesicles. The rate of cargo release is evaluated by tracking the changes in the fraction of encapsulated $S_{0}$ beads throughout the simulation (Figure 7c). The unilamellar vesicle shows a slightly lower release rate than the onion-like vesicle. About 97% of $S_{0}$-beads are released at timestep ${2.2\times 10}^{5}$ for onion-like vesicle, while about 94% of $S_{0}$-beads are released at timestep ${6.0\times 10}^{5}$ for unilamellar vesicle. Since the number of original $S_{0}$-beads is much larger for the unilamellar vesicle, a few $S_{0}$-beads can still be observed at timestep ${1.5\times 10}^{6}$. The faster release rate of the onion-like vesicle indicates that the membrane permeability of this vesicle is greater than that of the unilamellar vesicle.

### 3.4. Effect of Bulk Shear on Vesicle Behaviour

**Figure 8:**
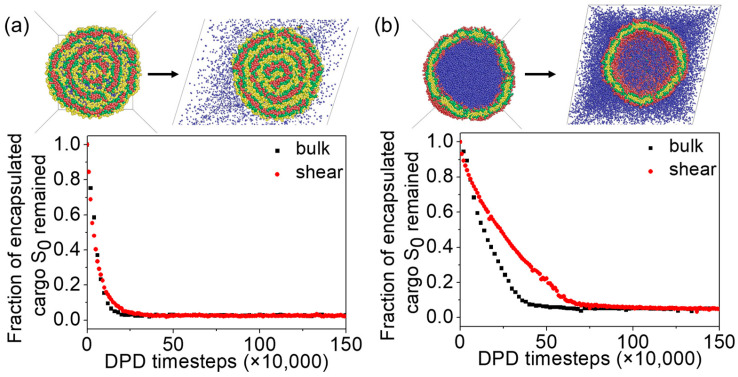
Vesicle release cargo under bulk shear: how the fraction of cargoes remaining inside vesicles changes with time for (**a**) onion-like vesicle and (**b**) unilamellar vesicle. Red, green, yellow, and blue beads correspond to C-, B-, A-, and S-beads, respectively.

It was previously noted in Section 3.3 that, in the absence of shear, both vesicles release cargoes, with the onion-like vesicles exhibiting a lower cargo release rate than the unilamellar vesicle. In this section, we aim to address the impact of shear on vesicle permeability. The effect of shear on cargo release is shown in Figure 8. Interestingly, the cargo release rate of the onion-like vesicle is almost unaffected by the application of shear (Figure 8a).

**Figure 9:**
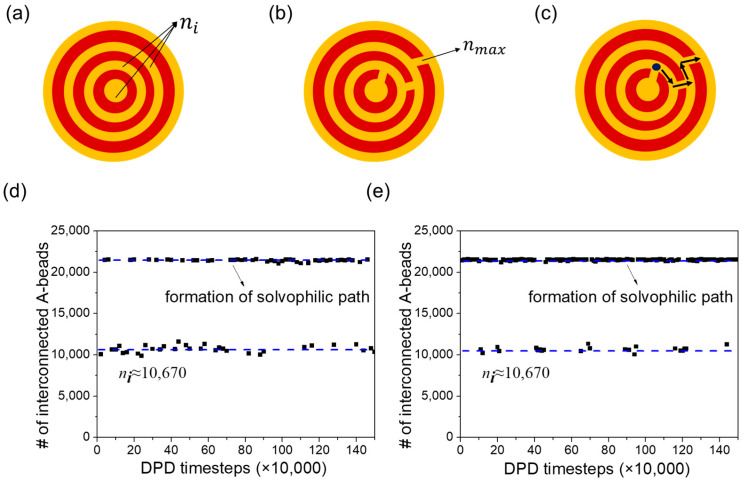
Schematics showing (**a**) no connection between the inner and outer A-bead shells, where $n=n_{i}$ is defined as the number of total interconnected hydrophilic A-beads constituting the inner shell of the onion-like vesicle, and (**b**) the inner and outer A-bead shells are linked, resulting in an increased number of interconnected A-beads, $n=n_{max}$. (**c**) A schematic showing the potential release path of cargoes. The number of interconnected A-beads starting from the inner shells for vesicles: (**d**) without shear and (**e**) under bulk shear. The blue dotted lines indicate the possible minimum and maximum number of interconnected A-beads in the vesicle.

The topology of the vesicle membranes under bulk shear can be monitored by counting the number of interconnected A-beads in the inner shell. Before applying shear, the number of interconnected A-beads in the entire inner shell of the onion-like vesicle was approximately $n_{i}\approx 10,670$ (Figure 9a). Changes in membrane topology can affect the quantity of interconnected A-beads. The point of maximum connectivity is reached with approximately 21,600 interconnected A-beads when the inner and outer A-bead shells are linked beads (Figure 9b). The numbers of interconnected A-beads, n, are presented in Figure 9c,d as functions of time before and after applying bulk shear to the onion-like vesicle. In the case without shear, the onion-like vesicles form a series of hydrophilic paths, which is why the vesicle release cargoes. In the case of shear, we observe the formation of hydrophilic paths more frequently compared with the no-shear case. However, unlike what we observed in raspberry vesicles, where the frequent formation of hydrophilic path promotes cargo release, in the case of onion-like vesicles, the cargo release rate is not significantly influenced by the frequency of hydrophilic path formation. This is because cargoes are distributed in different inner shells of the vesicles, and they need to traverse multiple paths to be released from the vesicle. So, even if the path forms frequently, if the beads do not transition smoothly to the next turn, they will not be released. Examining Figure 4b, we can see that the thickness of the hydrophobic membranes is only about 2.7 times the cutoff radius (${2.7\;r}_{c}$). This thin membrane structure makes it easier for hydrophilic beads to cross the membrane and to form the hydrophilic path.

**Figure 10:**
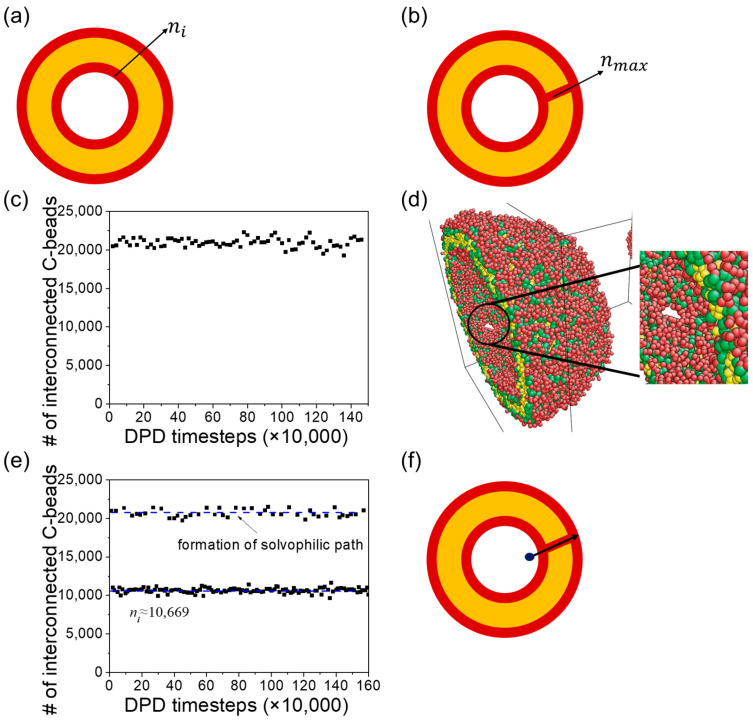
Schematics showing (**a**) no connection between the inner and outer C-bead shells, where $n=n_{i}$ is defined as the number of total interconnected hydrophilic C-beads constituting the inner shell of the unilamellar vesicle, and (**b**) the inner and outer C-bead shells are linked, resulting in an increased number of interconnected C-beads, $n=n_{max}$. (**c**) The count of interconnected C-beads originating from the inner shells for vesicles without shear. (**d**) Schematic showing the formation of pore in the unilamellar vesicle. (**e**) The count of interconnected C-beads originating from the inner shells for vesicles under shear. (**f**) A schematic showing the potential release path of cargoes. The blue dotted lines indicate the possible minimum and maximum number of interconnected C-beads in the vesicle.

Comparing the constant release rate without shear and with shear for the onion-like vesicle, we observed that the release rate for the unilamellar vesicle slows down under shear. The number of interconnected C-beads, denoted as n, is presented in Figure 10c,e, representing the changes over time before and after bulk shear is applied to the unilamellar vesicle. Before shear is applied, we found that the inner and outer C-bead shells were linked during the observation. Upon closer examination of the vesicle structure, we identified the existence of some pores in the vesicle’s membrane. These pores allowed for the release of cargoes, thereby enhancing the release rate. With the introduction of bulk shear to the vesicle, we noticed a reduction in the frequency of hydrophilic path formation. Upon careful examination of the vesicle, we discovered that the shear had a healing effect on the pores in the membrane. As a result, cargoes were released through the hydrophilic path across the membrane (Figure 10f), leading to a reduction in the release rate.

In summary, the encapsulation capacity of the unilamellar vesicle is higher than that of the onion-like vesicle. In vesicles under equilibrium conditions, initially encapsulated cargoes may be released due to membrane permeability. The onion-like vesicle releases cargoes through the formation of hydrophilic paths, while the unilamellar vesicle releases cargoes through pores. Shear can be employed to adjust the membrane structure of unilamellar vesicles, further modulating the cargo release rate.

**Figure 11:**
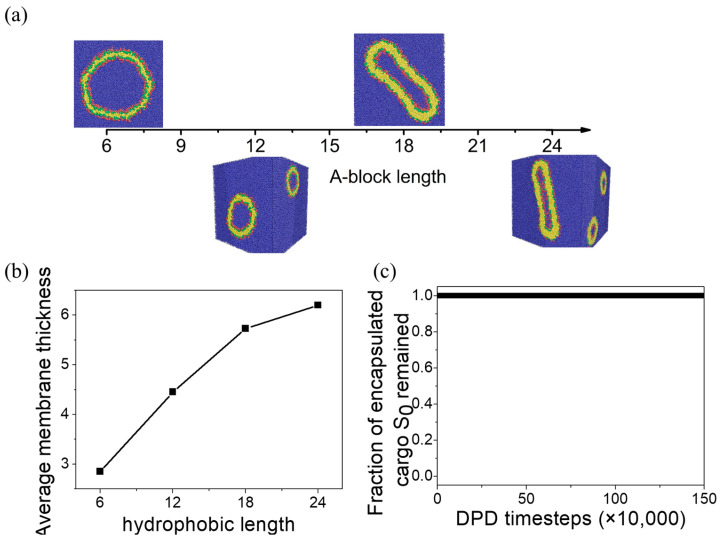
(**a**) Effect of A-block length on equilibrium aggregate structures. (**b**) Effect of A-block length on membrane thickness. (**c**) The fraction of original $S_{0}$-beads encapsulated inside vesicles at A-length $l_{A}=18$.

We infer that the formation of pores and hydrophilic paths in the unilamellar vesicles is observed due to the thin membrane. When the membrane is too thin, the hydrophilic blocks can easily cross the membranes, leading to frequent connections between the inner and outer hydrophilic shells. The permeability of the unilamellar vesicle under equilibrium can be regulated by increasing the length of the hydrophobic A-block in the polymer chains. Figure 11a presents the structural transition with varying A-block length in the case of $a_{AS}=65\;and\;a_{CS}=26$. In all cases of A-block length, unilamellar vesicles are obtained. The membrane thicknesses of the vesicles increase with the length of the hydrophobic A-block (Figure 11b). The number of originally encapsulated cargoes $S_{0}$ inside the vesicles remains constant during equilibrium simulations, suggesting that cargoes can be well protected by increasing the length of the hydrophobic block.

## 4. Conclusions

In this study, the CBABC pentablock terpolymer in S-bead solutions was used as the model system. With the assistance of DPD simulations, we observed a direct influence on the self-assembly behavior of C_3_B_3_A_6_B_3_C_3_ pentablock terpolymers in a selective solvent through the adjustment of block–solvent interaction strength. Various micelle and vesicle types are observed, including layered micelles, onion-like micelles, unilamellar vesicles, onion-like vesicles, and vesicle-in-vesicle structures, which depend on the $a_{AS}$ and $a_{CS}$ parameters. The phase diagram exhibits symmetry at $a_{AS}$ and $a_{CS}$ greater than $a_{BS}$, with layered micelles being prevalent in this region. Unilamellar vesicles are formed when the C-block is hydrophilic, while onion-like vesicles and vesicle-in-vesicle structures occur when the A-block is hydrophilic.

Polymeric vesicles are explored as potential nano-cargo carriers, focusing on their encapsulation efficiency and permeability. Utilizing DPD simulations, we delved into the detailed characterization of bulk properties and shear behavior of vesicles at specific time intervals. This approach allowed for a comprehensive understanding of the dynamic features and mechanical responses of the vesicle system. The unilamellar vesicle is more efficient at encapsulating solvent beads compared with onion-like vesicles. Both vesicles have permeable membranes under equilibrium conditions, allowing solvent exchange through hydrophilic channels or pores. Bulk shear is applied to investigate cargo release mechanisms in unilamellar and onion-like vesicles. Shear has no significant effect on the cargo release rate of onion-like vesicles, while it heals holes in unilamellar vesicles, resulting in a slower cargo release rate.

These findings offer insights into selecting CBABC vesicles as cargo carriers. To achieve vesicles with higher loading efficiency and lower permeability, pentablock terpolymers with hydrophilic end blocks are recommended. Additionally, membrane permeability can be adjusted by altering the length of hydrophobic blocks or by applying shear. In this study, the interaction parameters among blocks are set to be equal. It will be interesting to explore how varying the interaction parameters among blocks influences the formation of self-assembled morphologies, especially vesicles. Comparisons can then be made regarding cargo encapsulation ability and release behavior with the findings from the current work.

## Figures and Tables

**Table 1:** Interaction energy parameters $a_{ij}$.

Interaction Energy, $a_{ij}$ (in DPD Units)	A	B	C	S (Water)
A	25			
B	40	25		
C	40	40	25	
S (water)	26–105	45	26–105	25

## Data Availability

The data presented in this study are available from the corresponding author on request. The data are not publicly available due to privacy.
